# Perceived AI interactivity and Chinese EFL learners’ motivated learning behaviors: the moderating role of daily AI usage duration

**DOI:** 10.3389/fpsyg.2025.1689099

**Published:** 2025-11-19

**Authors:** Guo Min, Li Jiazhu

**Affiliations:** 1 Foreign Language Department, Xinyang Normal University, Xinyang, China; 2 English Education Department, Jeonbuk National University, Jeonju-si, Republic of Korea

**Keywords:** perceived AI interactivity, motivated learning behaviors, Chinese EFL learners, daily AI usage duration, moderation effect

## Abstract

The integration of Artificial intelligence (AI) tools in language education has reshaped learners’ perceptions and behavioral responses. While earlier studies have examined the cognitive and affective effects of learners’ perceived AI interactivity (PAII), less is known about its influence on motivated behavioral engagement. Motivated learning behaviors (MLBs) are crucial for sustained language learning. Accordingly, this study investigated how four dimensions of PAII predict MLBs among 171 Chinese English as a foreign language (EFL) learners, and whether daily AI usage duration (DAIUD) moderates these effects. Participants completed a validated questionnaire measuring PAII, MLBs, and DAIUD. Statistical results indicated that all four PAII dimensions significantly and positively predicted MLBs, with LC exerting the strongest predictive effect and R the weakest. Moreover, DAIUD significantly moderated the paths for R, LC, and P, strengthening their positive associations with MLBs, but it did not significantly moderate the LE path. This study expands the literature by applying a multidimensional construct of PAII to explain learners’ MLBs in Chinese EFL contexts. Moreover, these findings offer practical implications for designing learner-centered AI tools, and for educators providing tailored guidance for EFL learners in AI-supported learning.

## 1 Introduction

With the rapid advancement of educational technologies, various AI tools have been integrated into EFL education, such as AI chatbots (e.g., ChatGPT, Deepseek) and speech recognition systems (Laupichler et al., 2022; Xin and Derakhshan, 2025). These AI tools are increasingly favored because they provide timely and adaptive feedback regardless of time or location (Chen et al., 2025). As EFL learners engage more frequently with AI, their roles shift from passive recipients toward active participants in constructing knowledge (Huang et al., 2023; Luckin and Holmes, 2016; Wei, 2023). Previous studies have focused on technical functions of AI tools, overlooking the important role of the perceptions of AI interactivity in shaping learners’ behavioral engagement (Chien et al., 2025; Ma et al., 2025; Suriano et al., 2025). Therefore, this present research focused on EFL learners’ perceptions of AI interactivity within their learning contexts.

Perceived AI interactivity (PAII) refers to learners’ subjective evaluations of how responsive, adaptive, and engaging AI tools are during learning interactions. Four dimensions have been identified for this construct (Wang et al., 2025a), namely responsiveness (R), learner control (LC), learner engagement (LE), and personalization (P). Prior studies have revealed cognitive and affective influences linked to PAII, including improved learning achievement (Wang et al., 2023) and heightened enjoyment (Wang et al., 2025b). However, comparatively fewer investigations have addressed its impact on learning behaviors. Motivated learning behaviors (MLBs), characterized by persistence, sustained effort, and active exploration, are essential for success in AI-assisted learning (Reinders and White, 2011).

Grounded in Social Cognitive Theory (SCT), interactive contexts are expected to shape behavioral outcomes (Bandura, 1986; Wei, 2023). To further interpret why perceptions of AI interactivity promote learners’ behaviors, Self-Determination Theory (SDT) was selected. SDT posits that perceived interactivity satisfies psychological needs and may consequently influence behavioral regulation (Gagné and Deci, 2005). Moreover, the Unified Theory of Acceptance and Use of Technology (UTAUT) was used to examine how experiential factors influence the relationships. According to UTAUT, learning experience can shape the strength of perception-behavior relationships (Venkatesh et al., 2003).

Based on these theoretical frameworks, this study examines how the four PAII dimensions predict EFL learners’ MLBs and whether DAIUD moderates these effects. Two research questions were formulated: (1) How do the four dimensions of PAII predict EFL learners’ motivated learning behaviors? (2) How does daily AI usage duration moderate the predictive effect of PAII on learners’ motivated learning behaviors? Motivated learning behaviors (MLBs) refer to sustained effort, active exploration of resources, and participation and persistence in AI-assisted interactions. Meanwhile, daily AI usage duration (DAIUD) is defined as the average daily time learners spend using AI tools for academic purposes, measured on a 5-point frequency scale.

This study contributes to prior work by applying a multidimensional PAII framework to explain learners’ behavioral engagement in EFL learning contexts. This research provides practical implications for AI developers in designing interactive learning tools and for educators in creating tailored AI-supported contexts that foster meaningful engagement among less experienced learners.

## 2 Literature review

### 2.1 Artificial intelligence in language education

The integration of AI into education has reshaped how learners access, process, and construct knowledge (Laupichler et al., 2022; Xin and Derakhshan, 2025). In language education, tools such as automated writing evaluation, speech recognition, grammar correction, and AI-powered chatbots are widely used by English learners (Chen et al., 2025). T These tools are appreciated for their instant, adaptive feedback delivered in a flexible, learner-centered manner (Wei, 2023). Such features can promote higher levels of engagement (Huang et al., 2023). However, some scholars argue that the effectiveness of AI tools cannot be fully understood solely in terms of their technical affordances. Instead, learners’ subjective perceptions are crucial drivers of learning outcomes (Chien et al., 2025; Ma et al., 2025; Suriano et al., 2025). Consequently, gaining a deeper understanding of learners’ perceptions of AI interactivity is essential for a thorough evaluation of its educational impact.

### 2.2 Conceptualization of key variables

#### 2.2.1 Perceived AI interactivity

Perceived AI interactivity (PAII) refers to individuals’ subjective perception of their sense of control over the interaction process, as well as the responsiveness of AI tools (Shao and Chen, 2021). Unlike AI interactivity, which centers on the technical exchange between AI tools and users through prompts and feedback, PAII emphasizes learners’ subjective evaluations during the interaction. Based on this conceptualization, a validated scale was developed (Wang et al., 2025a), consisting of responsiveness (R), learner control (LC), personalization (P), and learner engagement (LE). Specifically, R captures the perceived timeliness and relevance of AI-supported feedback. LC reflects the extent to which learners can make decisions about navigation and content during AI interaction. LE encompasses emotional and cognitive involvement stimulated during the learning process. P denotes the degree to which AI feedback is adjusted to individual needs or prior knowledge. These four dimensions constitute the conceptual and measurement foundation of the research. However, previous studies have predominantly emphasized cognitive and affective outcomes, with much less attention paid to how PAII influences learners’ behavioral engagement, particularly within EFL contexts (Benvenuti et al., 2023; Kim et al., 2022; Lin et al., 2023).

#### 2.2.2 Motivated learning behaviors

Motivated learning behaviors (MLBs) are observable actions that reflect a learner’s willingness to initiate, sustain, and regulate learning in a goal-directed manner (Gagné and Deci, 2005). These behaviors represent the external demonstration of internal academic motivation (Schunk and DiBenedetto, 2020). Common examples include consistent effort, persistence when encountering difficulties, and proactive engagement in learning tasks. However, motivation itself is insufficient without the accompanying behavioral enactment. MLBs are considered essential for achieving long-term success in language learning, especially in self-regulated and AI-supported contexts (Reinders and White, 2011). Therefore, it is important to investigate how learners’ MLBs can be effectively promoted. Yet, only limited empirical work has explored the extent to which perceptions of AI interactivity predict MLBs in EFL environments. Accordingly, the present study examines how the four PAII dimensions predict EFL learners’ MLBs.

### 2.3 Theoretical framework

This study integrates Social Cognitive Theory (SCT), Self-Determination Theory (SDT), and the Unified Theory of Acceptance and Use of Technology (UTAUT) to explore how the four PAII dimensions shape learners’ MLBs and how AI usage experience (DAIUD) moderates these relationships. SCT (Bandura, 1986) provides a basis for the main effect, proposing that individuals’ behaviors are influenced by their cognitive interpretations of environmental variables. During AI-supported learning, PAII reflects learners’ interpretations of the interactive features of AI systems, including immediacy, adaptiveness, controllability, and engagement, which subsequently influence behavioral engagement (Song and Song, 2023). Moreover, SDT (Gagné and Deci, 2005) further clarifies the internal mechanisms linking PAII to MLBs, arguing that satisfaction of basic psychological needs (autonomy, competence, and relatedness) promotes sustained motivational engagement. Thus, the four dimensions of PAII are expected to facilitate MLBs by fulfilling these needs. Finally, UTAUT (Venkatesh et al., 2003) introduces the moderator of DAIUD. Then, UTAUT was employed to examine how participants’ learning experience using AI influence the relationships. According to UTAUT, learners with greater AI usage experience are more familiar with how to navigate the tools and leverage their functions. Hence, DAIUD, as a behavioral indicator of experience, is expected to shape the strength of the PAII–MLBs relationships.

### 2.4 Hypothesis development

#### 2.4.1 Perceived AI interactivity and motivated learning behaviors

Recent empirical research has examined how perceptions of AI interactivity are associated with learners’ behavioral engagement (Shao and Chen, 2021). Learners’ perceptions of timely and personalized responses from ChatGPT strengthened their sustained engagement in academic writing tasks, partly due to enhanced confidence and a heightened sense of competence (Song and Song, 2023). Likewise, students who perceived higher levels of interactivity, feedback quality, and control exhibited stronger self-regulation and persistence, as well as a greater sense of autonomy (Wei, 2023). In addition, qualitative findings have shown that learners’ perceptions of AI-related attributes, including usefulness, interactivity, and enjoyment, were positively connected to behavioral participation in AI-assisted learning (Wang et al., 2023). According to SDT (Gagné and Deci, 2005), the four PAII dimensions correspond to the three core psychological needs: R supports autonomy by providing timely reciprocal interaction; LC reinforces autonomy by enabling self-directed decision-making; LE enhances competence by sustaining cognitive involvement; and P contributes to relatedness through adaptive and individualized feedback. Based on both theoretical reasoning and prior evidence, four hypotheses were proposed:

*H*1: Responsiveness positively predicts EFL learners’ MLBs.

*H*2: Learner control positively predicts EFL learners’ MLBs.

*H*3: Learner engagement positively predicts EFL learners’ MLBs.

*H*4: Personalization positively predicts EFL learners’ MLBs.

Aside from the main constructs, previous research suggests that gender and English proficiency may also influence behavioral engagement in AI-assisted learning. Female learners often display stronger motivational engagement, whereas male learners tend to emphasize performance outcomes (Csizér and Kormos, 2008). Likewise, higher English proficiency is associated with greater self-efficacy and confidence when using tools, which may improve engagement (Wang and Wang, 2022). Therefore, to reduce potential confounding effects, gender and English proficiency were treated as control variables in this study.

#### 2.4.2 The moderation effect of DAIUD

Although the positive effects of PAII on MLBs are theoretically plausible, such effects may vary among different groups of learners. Prior studies indicate that learners with higher familiarity with AI are more capable of translating their perceptions of interactivity into motivated behaviors (Wang et al., 2025b; Wei, 2023). From the perspective of SDT, greater usage experience may strengthen familiarity with AI features, thereby enhancing motivational engagement (Huang et al., 2023; Wang et al., 2023). In UTAUT, DAIUD is conceptualized as a behavioral indicator of user experience that may moderate these associations. In this research, DAIUD refers to the amount of time learners spend using AI tools for academic activities each day. The following hypotheses were therefore formulated:

*H*5: DAIUD significantly moderates the relationship between responsiveness and MLBs.

*H*6: DAIUD significantly moderates the relationship between learner control and MLBs.

*H*7: DAIUD significantly moderates the relationship between learner engagement and MLBs.

*H*8: DAIUD significantly moderates the relationship between personalization and MLBs.

## 3 Methodology

To test the proposed hypotheses, a cross-sectional research design was employed using a self-reported online questionnaire.^1^ The study examined the predictive effect of PAII on EFL learners’ MLBs while considering the moderation role of DAIUD.

### 3.1 Subjects

A convenience sampling method was used to recruit 171 Chinese undergraduate English majors from a normal university in Henan Province, comprising 162 females and 9 males. Based on their English major test results, participants were divided into three proficiency groups: high (TEM-8, *N* = 7), intermediate (TEM-4, *N* = 103), and low (did not pass TEM-4, *N* = 61). Demographic information on gender and self-reported English proficiency was collected and subsequently treated as control variables in regression analyses.

All participants had about 1 year of experience using AI tools for academic learning, indicating a basic level of familiarity with AI-supported practices. Most reported using AI chatbots (e.g., Doubao, DeepSeek) and translation or grammar correction tools (e.g., Youdao, Grammarly) for academic purposes. Since all subjects were from a single institution and shared comparable educational backgrounds, the research context provided a controlled setting to examine perceived AI interactivity and MLBs. In addition, the gender distribution reflects the common demographic structure of English major cohorts in Chinese universities, where female students substantially outnumber males (Xu et al., 2023).

### 3.2 Research instruments

A structured questionnaire was used to investigate participants’ perceptions of AI interactivity, MLBs, and DAIUD. All constructs were measured (see Table A1) on a 5-point Likert scale ranging from 1 (strongly disagree) to 5 (strongly agree), with the exception of the DAIUD item, which adopted a 5-point frequency scale: 1 = “never,” 2 = “0 to 0.5 h per day,” and 5 = “more than 2 h per day.” The participants were asked to respond to the item, “*how long do you often use AI in learning each day?*” PAII was assessed using a 10-item scale adapted from prior work (Wang et al., 2025a), and MLBs were assessed using a 3-item scale adapted from earlier research (Wang and Wang, 2022). DAIUD reflects learners’ average daily time spent using AI tools for academic learning. The reliability and validity of the scale are presented below.

As shown in Table 1, Cronbach’s *α* values ranged from 0.55 to 0.88, whereas CR and AVE ranged from 0.58 to 0.85 and 0.52 to 0.70, indicating overall acceptable reliability and convergent validity. The lower reliability for the LC subscale (α = 0.53; CR = 0.58) is likely because of its two-item format, which often result in downward estimates of consistency (Cortina, 1993). However, its AVE (0.52) exceeded the minimum criterion of 0.50, reflecting satisfactory convergent validity (Hair, 2009; Hu and Bentler, 1999). The overall measurement model demonstrated acceptable fit (*χ*^2^/df = 2.21, CFI = 0.93, TLI = 0.90, GFI = 0.91, RMSEA = 0.08), and all standardized factor loadings were significant (*p* < 0.001). Although the RMSEA slightly surpassed the conventional cutoff of 0.08, it remains within the acceptable range for multifactor models (Browne and Cudeck, 1993; Hu and Bentler, 1999). Therefore, even though the original measures were not developed specifically for AI-supported contexts, their applicability to this EFL setting was empirically supported through CFA.

**Table 1:** Reliability and validity of the scale.

Dimensions	Items	Cronbach’s α	CR	AVE
R	3	0.83	0.85	0.70
LC	2	0.53	0.58	0.52
LE	3	0.80	0.81	0.66
P	2	0.70	0.70	0.62
MLBs	3	0.78	0.78	0.61
Overall scale	13	0.88	***—***	***—***

R, responsiveness; LC, learner control; LE, learner engagement; P, personalization; MLBs, motivated learning behaviors; CR, composite reliability; AVE, average variance extracted.

### 3.3 Data collection

Before data collection, all participants were informed of the study’s purpose and procedures. Informed consent was obtained from each respondent. Participation was voluntary and anonymous in accordance with ethical research guidelines. The data were collected in April 2025 during the spring semester. The questionnaire was administered online through the Wenjuanxing platform.^2^ To reduce environmental distractions, all participants completed the survey during scheduled class sessions under instructor supervision, requiring about five to 8 min. A total of 176 responses were returned. After removing patterned or careless submissions, 171 valid responses were retained.

### 3.4 Data analysis

The statistical analysis was conducted in SPSS 27.0 and AMOS 24.0. Prior to hypothesis testing, data were screened for missing values and examined for normality. The initial measurement contained 14 items for PAII and 4 items for MLBs. During CFA, items with standardized loadings below 0.50 were deleted (Hair, 2009), yielding a final measurement with 10 items for PAII and 3 for MLBs. Reliability and validity were then rechecked through Cronbach’s *α*, CR, and AVE, all of which met acceptable thresholds. Gender and English proficiency were included as control variables to minimize confounding effects (Csizér and Kormos, 2008; Wang and Wang, 2022). Independent samples t-tests and one-way ANOVA were used to examine whether gender and proficiency produced significant group differences. Pearson correlation analysis was conducted to explore relationships among PAII, MLBs, and DAIUD.

To test the hypotheses, hierarchical multiple regression analyses were carried out. Control variables were entered into Step 1, followed by the four dimensions of PAII in subsequent steps. Finally, moderation analyses were employed to analyze the interaction effects between each dimension of PAII and DAIUD on MLBs. Simple slope analyses conducted (+1 SD) and low (−1 SD) levels of DAIUD to interpret significant interactions.

## 4 Results

### 4.1 Descriptive statistics and demographic differences

To determine whether learners’ demographic characteristics (gender and English proficiency) affected the principal study variables, independent-samples *t*-tests and one-way ANOVA were conducted.

#### 4.1.1 Gender differences

Independent-samples *t*-tests revealed no statistically significant gender differences across any of the study variables (*p* > 0.05; see Table 2, Panel A). Male (*n* = 9) and female learners (*n* = 162) reported comparable levels of PAII, MLBs, and DAIUD. Although the mean scores for R, LC, LE, and P were slightly higher among female learners, the differences were not statistically meaningful. Thus, gender did not exert a significant effect on learners’ PAII, MLBs, or DAIUD.

**Table 2:** Group differences in subjects’ gender and English proficiency.

Panel A: Gender differences (independent sample *T*-tests)						
Variable	Male (*n* = 9)		Female (*n* = 162)		*t*	*p*
	Mean	SD	Mean	SD		
R	3.56	0.44	3.68	0.66	−0.54	0.59
LC	3.39	0.70	3.48	0.62	−0.42	0.67
LE	3.41	0.57	3.55	0.56	−0.72	0.47
P	3.61	0.22	3.74	0.54	−1.56	0.14
MLBs	3.33	0.47	3.52	0.53	−1.06	0.29
DAIUD	3.00	1.12	2.83	0.87	0.55	0.58

Panel B: English proficiency differences (one-way ANOVA analysis)								
Variable	Low (*n* = 61)		Medium (*n* = 103)		High (*n* = 7)		*F*	*P*
	Mean	SD	Mean	SD	Mean	SD		
R	3.67	0.56	3.66	0.71	3.81	0.50	0.17	0.85
LC	3.47	0.64	3.49	0.62	3.36	0.38	0.15	0.87
LE	3.43	0.58	3.60	0.55	3.62	0.41	1.75	0.18
P	3.69	0.51	3.77	0.55	3.71	0.39	0.42	0.66
MLBs	3.52	0.53	3.50	0.53	3.71	0.41	0.55	0.58
DAIUD	2.87	0.88	2.80	0.83	3.14	0.79	0.55	0.58

No statistically significant differences were found (*p* > 0.05). SD, standard deviation; R, responsiveness; LC, learner control; LE, learner engagement; P, personalization; MLBs, motivated learning behaviors; DAIUD, daily AI usage duration.

#### 4.1.2 English proficiency differences

A one-way ANOVA was conducted to explore whether different proficiency levels (low, medium, high) were associated with variations in PAII, MLBs, or DAIUD (see Table 2, Panel B). No statistically significant differences were observed among the three proficiency groups (*p* > 0.05). Although higher proficiency learners tended to report marginally greater mean scores, these differences were not statistically significant. Consequently, English proficiency did not significantly influence PAII, MLBs, or DAIUD.

In summary, neither gender nor English proficiency exerted a statistically significant influence on PAII, MLBs, or DAIUD. These findings suggest that demographic variables did not introduce systematic bias into the main constructs, thereby providing a solid foundation for subsequent analyses.

### 4.2 Correlation analysis

Pearson correlation analysis was conducted to examine the relationships among the core study variables (see Table 3). The results indicated that all four PAII dimensions were significantly and positively correlated with MLBs (*r* = 0.27–0.64, *p* < 0.01), implying that higher levels of perceived interactivity were associated with stronger motivated learning behaviors. Furthermore, the four dimensions of PAII were positively intercorrelated (*r* = 0.39–0.65, *p* < 0.01), reflecting satisfactory internal coherence among the subscales. Consequently, all four dimensions were deemed appropriate for inclusion in the hierarchical regression analysis to assess their combined predictive effect on MLBs. However, DAIUD was not significantly correlated with any of the main variables (*p* > 0.05), indicating that its role may be better understood as a moderator rather than a direct predictor.

**Table 3:** Statistical result of Pearson correlation analysis (*N* = 171).

	Mean	SD	DAIUD	MLBs	R	LC	LE	P
DAIUD	2.84	0.88	1					
MLBs	3.67	0.65	0.11	1				
R	3.47	0.62	0.11	0.57**	1			
LC	3.54	0.56	0.10	0.39**	0.47**	1		
LE	3.52	0.53	0.13	0.27**	0.33**	0.64**	1	
P	3.74	0.53	0.09	0.42**	0.43**	0.65**	0.51**	1

*p < 0.05, **p < 0.01. SD, standard deviation; R, responsiveness; LC, learner control; LE, learner engagement; P, personalization; MLBs, motivated learning behaviors; DAIUD, daily AI usage duration.

### 4.3 Hierarchical regression analysis

Before conducting the regression analysis, multicollinearity was assessed using the variance inflation factor (VIF) and tolerance values. The VIFs ranged from 1.00 to 1.91 and tolerance values from 0.52 to 0.10, all within acceptable thresholds (VIF < 10; Tolerance > 0.20) (Hair, 2009), indicating no multicollinearity issues.

Hierarchical regression was then employed to evaluate the predictive effects of the four PAII dimensions on MLBs. As shown in Table 4, in Model 1 the control variables and DAIUD jointly explained 2.6% of the variance in MLBs but did not reach statistical significance (*F* (3,167) = 1.48, *p* = 0.22 > 0.05). In Model 2, the inclusion of R significantly improved the model (Δ*R*^2^ = 0.06, *p* = 0.004 < 0.01), indicating a significant positive contribution (*β* = 0.25, *p* = 0.001 < 0.01). In Model 3, adding LC further increased the explained variance (Δ*R*^2^ = 0.04, *p* = 0.01 < 0.01). Model 4 demonstrated a substantial improvement when LE was entered (Δ*R*^2^ = 0.29, *p* < 0.001), with LE emerging as the strongest predictor (*β* = 0.63, *p* < 0.001). Finally, Model 5 showed that including P yielded a smaller yet still significant increase in variance explained (Δ*R*^2^ = 0.02, *p* = 0.04 < 0.05), reflecting a positive predictive effect (*β* = 0.17, *p* = 0.04 < 0.05). In total, the full regression model accounted for 43.3% of the variance in MLBs (*R*^2^ = 0.43).

**Table 4:** Statistical result of hierarchical regression analysis (*N* = 171).

Predictor	Model 1	Model 2	Model 3	Model 4	Model 5
Step 1: Control variables and moderator					
Gender	0.09	0.08	0.07	0.05	0.04
English proficiency	0.04	0.03	0.03	−0.06	−0.05
Moderator					
DAIUD	0.14	0.11	0.10	0.06	0.06
Step 2: Four dimensions of PAII					
R	—	0.25**	0.11	0.003	−0.02
LC	—	—	0.25**	0.02	0.01
LE	—	—	—	0.63***	0.54***
P	—	—	—	—	0.17*
Model summary					
*R* ^2^	0.03	0.09	0.13	0.42	0.43
Δ*R*^2^	—	0.06**	0.04**	0.29***	0.02*
*F*	1.48	4.00**	4.93***	19.63***	17.80***

Standardized *β* coefficients are reported. *p < 0.05, **p < 0.01, ***p < 0.001. R, responsiveness; LC, learner control; LE, learner engagement; P, personalization; MLBs, motivated learning behaviors; DAIUD, daily AI usage duration. *R*^2^, explained variance; Δ*R*^2^, change in explained variance from the previous model.

These findings indicate that, after controlling for gender and English proficiency, all four PAII dimensions significantly contributed to the prediction of MLBs. LE exerted the strongest predictive influence, followed by LC, P, and R. This pattern aligns with SCT and SDT, which highlight that perceptions of autonomy, competence, and responsive feedback collectively promote learners’ MLBs.

### 4.4 Moderation analysis

Moderation analysis was conducted to further examine whether DAIUD moderated the relationships between the four PAII dimensions and MLBs. As shown in Table 5, three out of the four interaction effects reached statistical significance, indicating that DAIUD partially moderated these relationships. Specifically, the interaction between LC and DAIUD was the strongest (*β* = 0.20, *p* = 0.01 < 0.05, Δ*R*^2^ = 0.03), suggesting that learners with longer AI usage duration derived more motivated behaviors from a heightened sense of control in AI-supported learning. The interaction between R and DAIUD was also significant (*β* = 0.15, *p* = 0.001 < 0.01, Δ*R*^2^ = 0.02), implying that learners with higher DAIUD benefited more from timely AI feedback. Likewise, the interaction between P and DAIUD was significant (*β* = 0.15, *p* = 0.008 < 0.05, Δ*R*^2^ = 0.015), showing that greater AI usage strengthened the contribution of adaptive and individualized feedback to MLBs.

**Table 5:** Statistical result of moderation analysis (*N* = 171).

Interaction term	*β*	*t*	Δ*R*^2^	*p*
R × DAIUD	0.15	2.14	0.02	0.001**
LC × DAIUD	0.20	2.96	0.03	0.01*
LE × DAIUD	0.07	1.19	0.01	0.23
P × DAIUD	0.15	2.05	0.02	0.008**

*p < 0.05, **p < 0.01. All variables were mean-centered. R, responsiveness; LC, learner control; LE, learner engagement; P, personalization.

However, the moderating effect of DAIUD on the relationship between LE and MLBs did not achieve statistical significance (*β* = 0.07, *p* = 0.23 > 0.05, Δ*R*^2^ = 0.01). This finding suggests that although LE is positively associated with MLBs, its predictive strength remains relatively stable regardless of daily usage duration.

**Figure 1:**
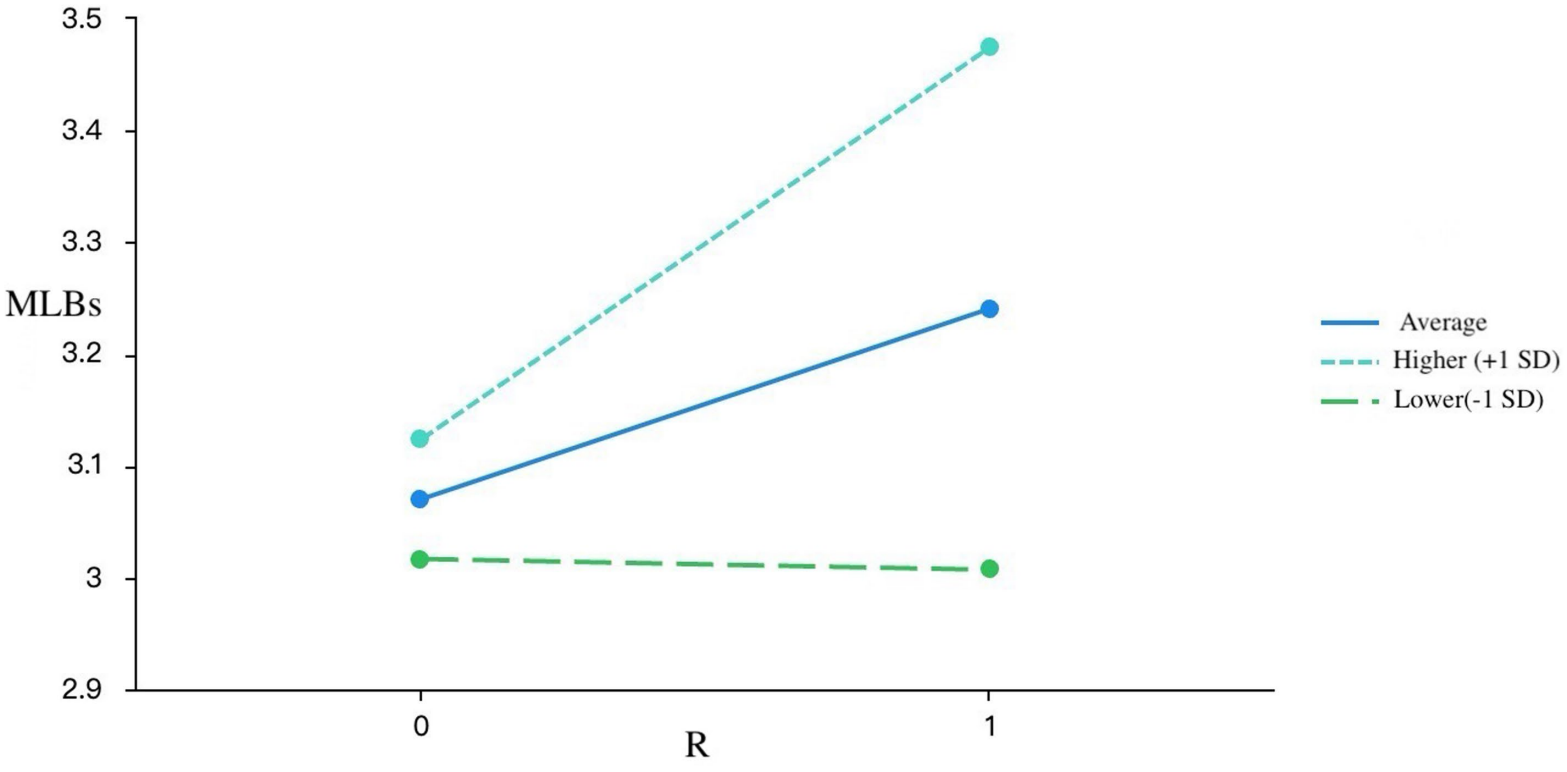
The shaded areas represent 95% confident intervals. *R*, responsiveness; MLBs, motivated learning behaviors *N* = 171.

**Figure 2:**
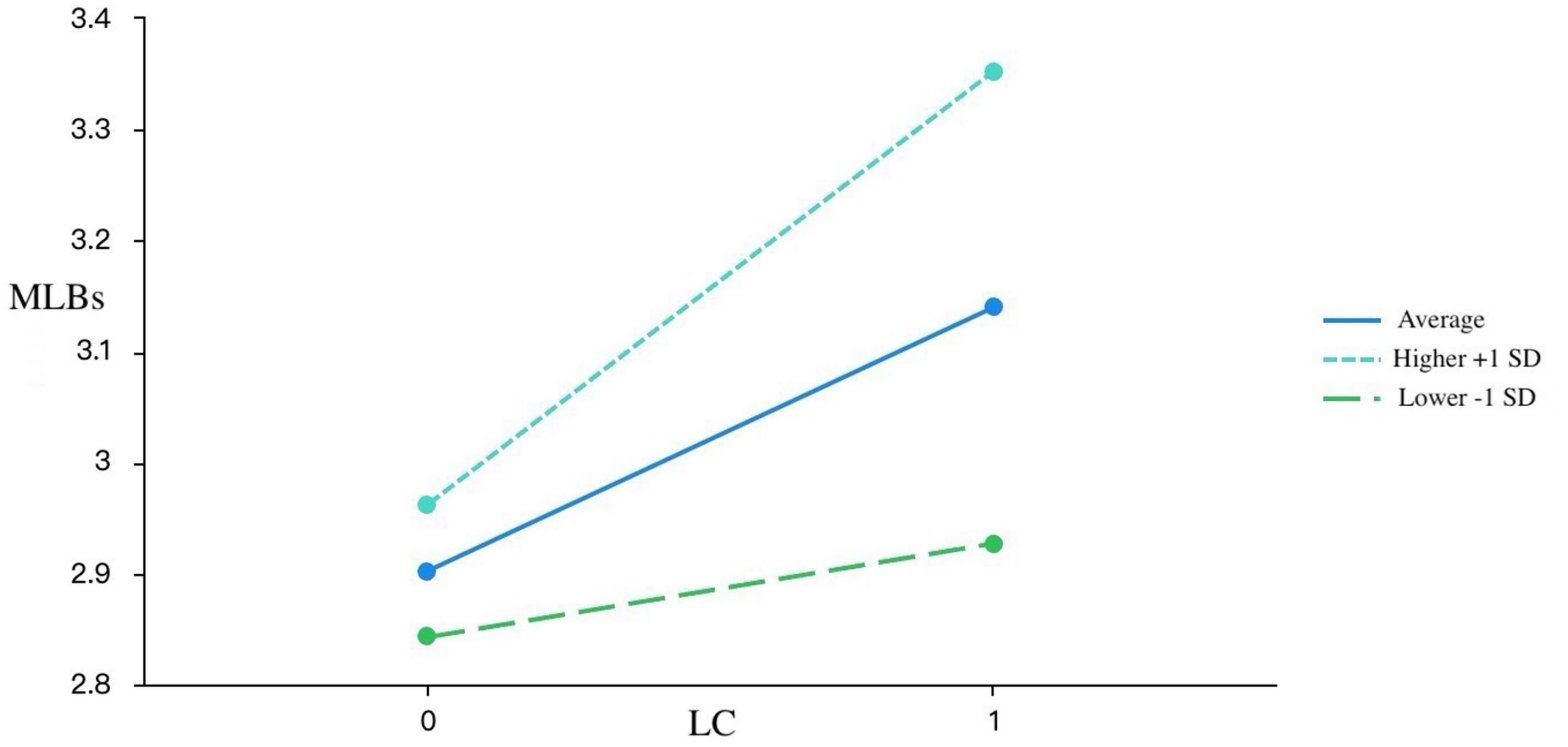
The shaded areas represent 95% confident intervals. *LC*, learner control; MLBs, motivated learning behaviors *N* = 171.

**Figure 3:**
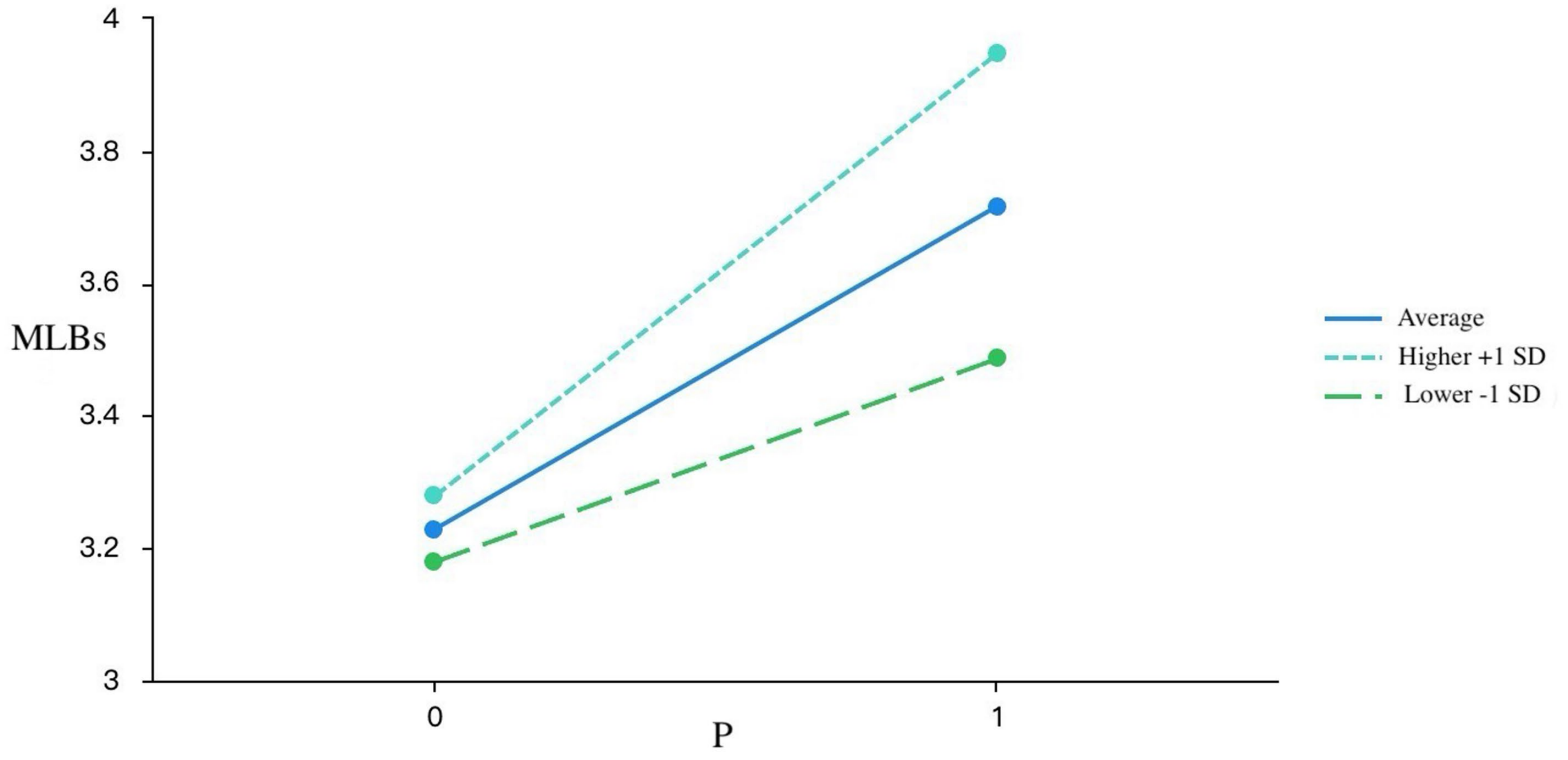
The shaded areas represent 95% confident intervals. *P*, Personalization; MLBs, motivated learning behaviors *N* = 171.

Simple slope analyses (Figures 1–3) confirmed the moderation effects of DAIUD on R, LC, and P. For learners with low DAIUD (−1 SD), the slopes between PAII and MLBs were relatively flat, whereas for those with high DAIUD (+1 SD), the slopes became markedly steeper. Specifically, for R, the simple slope was non-significant among low-AI-usage learners (SE = 0.09, *t* = −0.11, *p* = 0.91 > 0.05), but became significant and stronger among high-AI-usage learners (SE = 0.07, *t* = 4.80, *p* < 0.001). A similar pattern emerged for LC: the effect on MLBs was not significant for learners with low DAIUD (SE = 0.10, *t* = 0.89, *p* = 0.38 > 0.05), yet was significant and steeper for those with high DAIUD (SE = 0.08, *t* = 4.92, *p* < 0.001). For P, the relationship with MLBs was significant for both low and high DAIUD groups but was considerably stronger among high-AI-usage learners (low: SE = 0.10, *t* = 3.20, *p* = 0.002 < 0.01; high: SE = 0.10, *t* = 7.29, *p* < 0.001).

## 5 Discussion

This study examined how the four PAII dimensions predict Chinese EFL learners’ MLBs, with DAIUD as a moderator. The findings showed that all four PAII dimensions significantly predicted MLBs, with LE reflecting the strongest predictive power. Besides, DAIUD partially moderated the effects by strengthening the influence of R, LC, and P, but not that of LE.

For the first research question, the results can be interpreted through Social Cognitive Theory (Bandura, 1986) and Self-Determination Theory (Gagné and Deci, 2005). Responsive, controllable, engaging, and personalized AI environments enhance learners’ competence (Huang et al., 2023), autonomy (Ma et al., 2025), and relatedness (Song and Song, 2023; Wei, 2023). Subsequently, the effects can foster learners’ motivational patterns in AI-supported learning. Notably, LE showed the strongest predictive power among four dimensions, echoing prior literature that emotionally engaging interactions with AI tools most effectively sustain persistence and task investment (Wang et al., 2023; Wang and Wang, 2022; Wei, 2023). This supports the perspective of Flow Theory, immersion and enjoyment transform cognitive engagement into positive behaviors. In contrast, R exerted a relatively weaker effect, consistent with previous proposition (Chien et al., 2025), who argued that immediacy of AI tools alone failed to maintain individuals’ behavioral engagement without deeper autonomy support or affective resonance.

The moderation findings further indicated that DAIUD strengthened the effects of R, LC, and P on MLBs. This suggests that learners who use AI tools more extensively are better able to convert perceived interactivity into motivational outcomes. In line with UTAUT (Venkatesh et al., 2003), increased AI usage enhances familiarity with AI tools’ features, thereby amplifying the motivational impact of responsive feedback and adaptive support (Huang et al., 2023; Wang et al., 2023).

However, DAIUD did not moderate the effect of LE. Based on Flow Theory (FT) (Csikszentmihalyi, 1990), which conceptualizes engagement as an optimum state rather than an accumulative trait. The learners’ spending more time on AI interaction each day may experience habituation and reduced novelty (Barthelmäs and Keller, 2021; Keller and Bless, 2008). However, occasional learners can achieve remarkable engagement by novel experiences (Barthelmäs and Keller, 2021; Peifer and Engeser, 2021). Hence, the motivational influence of LE remained stable regardless of usage duration, indicating that the quality of engagement outweighs the quantity of AI exposure.

This study contributes in two primary ways. First, it extends prior research by moving beyond cognitive and affective outcomes to examine how PAII relates to learners’ behavioral engagement. Second, it offers culturally grounded insights into EFL learners’ motivational patterns in AI-supported contexts.

Notwithstanding its contributions, this study has limitations. First, DAIUD was measured with a single self-reported item, which may limit its reliability. Although this item can capture learners’ time spent on AI-supported learning, it might not fully reflect the multidimensional nature of AI interaction, such as the cognitive intensity of AI usage. Second, the single-site sample with a gender imbalance restricts the generalizability of the findings. Finally, the cross-sectional design of this study failed to capture the dynamic changes in the relationship between learners’ perceptions of AI interactivity and motivated learning behaviors.

Therefore, future studies could employ multi-item or behavioral log-based measures to more accurately capture learners’ daily AI usage and reduce self-report bias. In addition, future research should seek more gender-balanced samples or conduct multi-group analyses to examine potential gender differences. Moreover, longitudinal designs with more diverse institutional contexts will be valuable for validating and extending current findings.

## 6 Conclusions and implication

This study examined how PAII predicts Chinese EFL learners’ MLBs, with DAIUD serving as a moderator. All four PAII dimensions significantly predicted MLBs, with LC showing the strongest effect and responsiveness the weakest. DAIUD significantly moderated the effects of R, LC, and P, indicating that learners who used AI tools more frequently were more capable of transforming their perceptions of interactivity into motivated behaviors. However, DAIUD did not moderate the LE effect, suggesting that affective immersion is more qualitative than cumulative. Overall, the findings imply that the benefits of PAII depend more on the meaningfulness and depth of interaction rather than the sheer volume of daily usage. Theoretically, the findings extend motivational research by integrating SCT, SDT, UTAUT, and FT to explain how perceptions of AI interactivity predict behavioral engagement. Practically, these results suggest that AI developers should prioritize learner control, engagement, and personalization rather than mere efficiency. Educators should likewise provide differentiated scaffolding, particularly for learners with limited AI familiarity, to strengthen meaningful participation in AI-supported learning environments.

## Data Availability

The raw data supporting the conclusions of this article will be made available by the authors, without undue reservation.

## Footnotes

^1^ https://www.wenjuan.com

^2^ https://www.wenjuan.com

## Appendix

**Table A1:** Questionnaire.

**Dimensions**		**Items**
PAII	R	AI processes my response quickly.
		AI is fast in responding to my questions.
		I can communicate with AI directly for further questions.
	LC	While using AI, I choose freely what I want to learn.
		While using AI, my actions decide the kind of my learning experiences.
	LE	AI can keep my attention when interacting with them in learning.
		I am involved in the learning experiences using AI.
		I have an enjoyable feeling while using AI in learning.
	P	AI satisfies my specific learning needs.
		AI takes my needs as its own preferences.
MLBs		After using AI, I actively look for more information related to the learning content.
		After using AI, I still persist in completing learning tasks even though they are challenging.
		Interacting with AI motivates me to take initiative in my learning.
DAIUD		How long do you often use AI in learning each day?

## Glossary

AI: Artificial Intelligence
EFL: English as a Foreign Language
PAII: Perceived AI Interactivity
R: Responsiveness
LC: Learner Control
LE: Learner Engagement
P: Personalization
MLBs: Motivated Learning Behaviors
DAIUD: Daily AI Usage Duration
TEM: Test for English Majors
SCT: Social Cognitive Theory
SDT: Self-Determination Theory
UTAUT: Unified Theory of Acceptance and Use of Technology
FT: Flow Theory
AMOS: Analysis of Moment Structures (statistical software)
SPSS: Statistical Package for the Social Sciences (statistical software)
CFA: Confirmatory Factor Analysis
CR: Composite Reliability
AVE: Average Variance Extracted
CFI: Comparative Fit Index
TLI: Tucker–Lewis Index
GFI: Goodness of Fit Index
RMSEA: Root Mean Square Error of Approximation
VIF: Variance Inflation Factor
SD: Standard Deviation
